# Supplementary material for: Splice-Junction-Based Mapping of Alternative Isoforms in the Human Proteome
Source: Cell Rep. Author manuscript; Available in PMC 2020 Jan 15. (PMC6961840; doi:10.1016/j.celrep.2019.11.026)
Supplement: 2 [file NIHMS1546469-supplement-2.zip › DF1/PXD010154/Esophagus/Esophagus_10_SVIL_RPELCTSHSETPTVDDEEK.pdf]

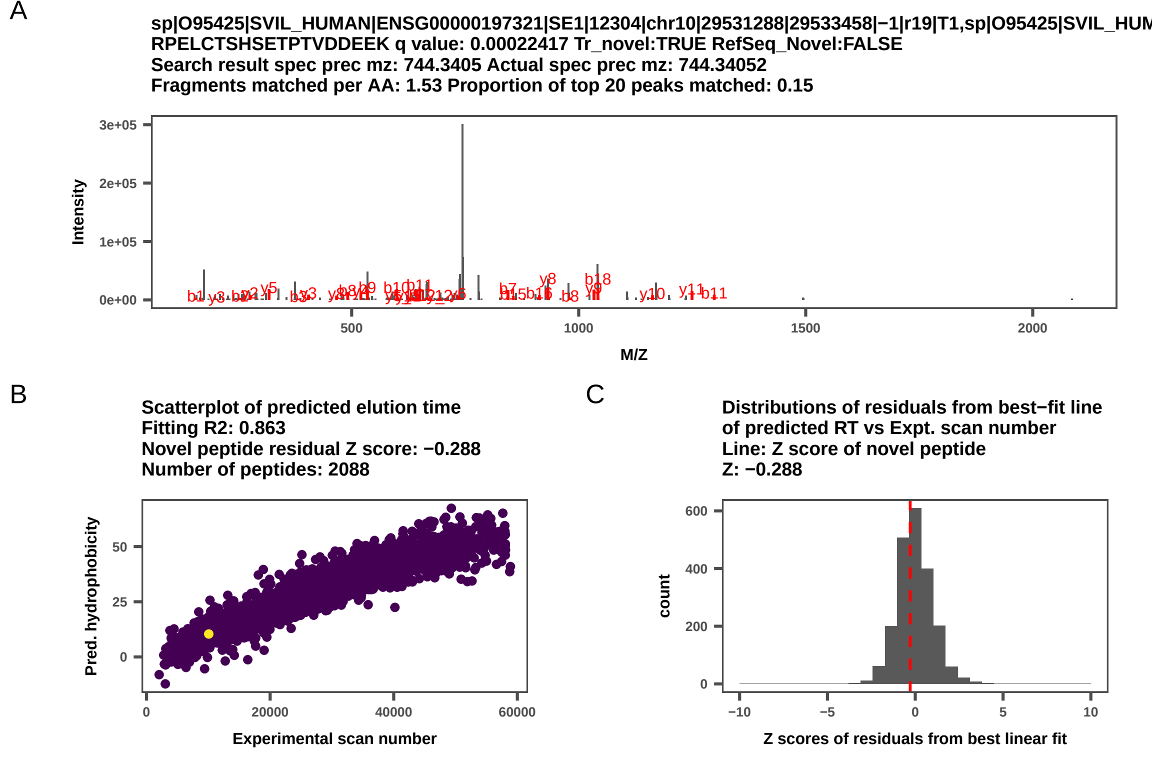
A
sp|O95425|SVIL_HUMAN|ENSG00000197321|SE1|12304|chr10|29531288|29533458|−1|r19|T1,sp|O95425|SVIL_HUM
RPELCTSHSETPTVDDEEK q value: 0.00022417 Tr_novel:TRUE RefSeq_Novel:FALSE
Search result spec prec mz: 744.3405 Actual spec prec mz: 744.34052
Fragments matched per AA: 1.53 Proportion of top 20 peaks matched: 0.15
Intensity
3e+05
2e+05
1e+05
0e+00
500
1000
1500
2000
M/Z
b1
y5
y8
b18
y10
y11
b11
b8
b7
B
Scatterplot of predicted elution time
Fitting R2: 0.863
Novel peptide residual Z score: −0.288
Number of peptides: 2088
Pred. hydrophobicity
50
25
0
0
20000
40000
60000
Experimental scan number
C
Distributions of residuals from best−fit line
of predicted RT vs Expt. scan number
Line: Z score of novel peptide
Z: −0.288
count
600
400
200
0
−10
−5
0
5
10
Z scores of residuals from best linear fit
